# On Idiopathic Anæmia

**Published:** 1863-10

**Authors:** S. O. Habershon

**Affiliations:** M. D., London F. R. C. P., Senior Assistant-Physician to, and Lecturer on Materia Medica at, Guy’s Hospital


### SELECTED.

### ON IDIOPATHIC ANÆMIA.

By S. O. HABERSHON, M. D., Lond., F. R. C. P.,

*Senior Assistant-Physician to, and Lecturer on Materia Medica at, Guy's Hospital.*

In some of those morbid conditions with which we are most familiar, it is as difficult to trace the source of the earlier symptoms as in others where the pathology is acknowledged to be more obscure. The symptoms of chlorosis are familiar, and it is a state recognized with great facility, and one of those in which right treatment soon manifests a most beneficial result; but the source of the changed condition of the blood so constant in this disease is not known. The pallor and the sense of weakness, the cessation of menstruation or its non-commencement, are preceded by defective nutrition and by impaired or capricious appetite, and the changes in the blood itself appear to be secondary to the imperfect absorption and assimilation of food. The climacteric modifications in the condition of the pelvic viscera, accompanied by turgescence in the vascular supply of the ovaries, are simultaneous with other developmental changes, not only in the glandular organs, but in the whole nervous system; and a morbid condition of the sympathetic or vaso-motor nerve is coincident with, if not causative of, chlorisis. And although the visceral nerve supply may be modified by mental impressions, and amenorrhœa may be caused by nervous shock, more frequently chlorisis and amenorrhœa are slowly induced by defective nutrition.

As to this state of chlorisis, there are degrees of severity; in some it is a transient malady; in others it is very protracted, and, as to its result, of serious import. When chlorisis becomes permanent, other degenerative changes take place; either the deposition of strumous product in the abdomen, or in the lungs, or in the brain follows, or simple fatty degeneration results, or both states may be conjoined.

The following case of anæmia was closely allied to some of the instances of protracted chlorisis which occasionally occur. Several such cases have been recorded during the last few years and theories have been put forth as to their true character; but, whilst they naturally are divided according to the organ especially diseased, and which has apparently led to a modified state of blood, there are some instances in which as yet no source of disease can be traced, and in our ignorance we are compelled to use the term "idiopathic anæmia." Thus an anæmic condition has been attributed—1st, to chronic diseases of a suppurating character, as some diseases of the spine, or to anything constituting a constant exhausting drain upon the system, hyper-lactation; 2nd, to the effect of acute disease, as rheumatic fever; 3d, to diseases of the spleen, as in leuco-cythæmia; 4th, to general disease of the lymphatic glands; 5th, to strumous cachexia; 6th, to miasmatic poisoning, ague, with which enlargement of the spleen is also associated; 7th, to disease of the supra-renal capsules; 8th, to actual loss of blood—internal or external hæmorrhage; 9th, to the effect of mercurialization; 10th, to the excessive use of alkalies, or the drinking of large quantities of water; 11th, to cancerous cachexia; 12th, to chronic ulceration of the stomach, and dyspepsia; 13th, to some diseases of the kidneys, as abluminuria; 14th, to insufficient light, with improper food and impure air; 15th, to chlorosis. But in some cases, although closely allied to chlorotic mal-nutrition, no source of disease can be detected. The patient's strength fails, pallor of the countenance ensues, and atrophy slowly advances, till the exhaustion becomes extreme. The instance before us was of this class, and the fatal degeneration of the heart and serous effusion into the pericardium were merely indications of advanced mal-nutrition, and they became the immediate cause of death.

The causes of anæmia which we have enumerated may, however, be divided into three classes—1st. Causes interfering with the proper reformation or the renewal of the blood, as diseased glands, mesenteric or lymphatic, &c. 2d. Causes operating directly upon the blood itself, as the effect of physical agents, mercury, water, alkalies; so also the actual loss of blood in the different forms of hæmorrhage; here also we may mention albuminuria, and perhaps ague poison. 3d. Excessive waste, the demand upon the nutritive power of the system being greater than the compensative supply; thus although the blood may be reformed properly, that renewal is not sufficient to compensate for the constant loss which takes place from exhaustive discharges.

Where shall we place struma and chlorosis? If we attribute these diseases to imperfect assimilation of food, and to a primary defect in the digestive organs, are we correct in placing them in the first category? Again, to what extent does the nervous system, in its sympathetic or vaso-motor nerve, influence nutritive changes? Fright and mental distress interfere with the digestion and absorption of food by means of the connexion between the cerebro-spinal and the sympathetic nerves. But, may this retarding influence be permanent; and may we not correctly attribute some forms of anæmia to diminished power in the sympathetic centres rather than to the organs which they influence? These questions naturally force themselves upon the mind in the consideration of a case such as the following, and although we would seek to be on our guard lest we hide our ignorance under a name, still something is gained if we can ascertain the direction of the origin of the disease.

*Idiopathic anæmia, commencing with gastric irritation; fatty degeneration of the heart.*—Eliza W——, aged forty, was admitted into Guy's Hospital on September 25th, 1861, under

Dr. Habershon's care. She was a single woman, who had resided at Rotherhithe, and obtained her livelihood by her needle. For six years menstruation had ceased, and during the latter four years she had felt stronger. Of delicate general health, and of spare and anæmic appearance, she had for many years suffered from occasional attacks of vomiting; but for eight months these attacks of vomiting had been of almost daily occurrence, with an occasional omission of a week. The vomiting sometimes came on directly after food; at other times there was an interval of many hours. There was pain down the sternum to the scrobiculus cordis, of an aching character, and increased by food and by vomiting; a burning sensation, amounting to pain, in the shoulders and back, troubled her; and also a "dragging pain" in the joints. The bowels were sometimes much relaxed, at other times confined for several days. There had been no hæmatemesis, but she thought that a little blood had been ejected three or four months before admission. She was blanched and emaciated; the chest was normal, and she had not any cough; the abdomen was contracted, and there was tenderness at the serobiculus cordis,but no dullness, and no pain in the back; the tongue was clean; the pulse compressible; the feet not swollen; the urine was rendered slightly turbid by heat, but cleared by nitric acid, and there was slight mucous deposit. Ordered, compound iron pill, two grains; creosote, half a minim; extract of henbane, two grains; three times a day. To take chop and four ounces of sherry wine; unable to take milk or beef tea.

September 28th.—Complained much of sickness and pain, and was ordered nitrate of bismuth, ten grains; carbonate of soda, ten grains; chloric ether, ten minims, in mucilage mixture.

On October 2nd, she complained of the medicine burning her throat. The roof of the mouth was granular, but nothing could be seen in her throat. She was very anæmic, but suffered less from vomiting. The medicine was ordered to be diluted; but as she still complained much of it, it was changed for bismuth, soda and conium. The offensive smell became then the great objection.

Oct. 19th.—Lime water with milk was ordered, and half an ounce of quinine mixture (one grain) given three times a day. On Dec. 3d, Dr. Barlow prescribed one-twelfth of a grain of strychnia with dilute phosphoric acid three times a day. This medicine did not agree; a sense of exhaustion, with a severe pain in the abdomen, came on. The pain became so severe, and the prostration so suddenly extreme, that it was doubted whether perforation had taken place. The pulse at the wrist was scarcely perceptible. A poppy poultice was applied to the abdomen, and ten minims of the solution of hydro-chlorate of morphia given in camphor mixture. On January 1st, 1862, she had greatly rallied, and there had been no return of pain. She was able to take food, such as jelly, &c., and sometimes a chop. The bowels were constipated. She was still very much blanched, but less distressed, and her countenance less haggard. On the 7th she had improved in health; and her appetite had so increased that she quite enjoyed her food. On the 15th she was able to sit up, and she slowly improved so as to leave the hospital.

She was re-admitted in the autumn (October 4th) of 1862, blanched, emaciated and extremely feeble. No evidence of disease of the stomach could be satisfactorily found; no pain after food; no vomiting; no tumor; no distension of the abdomen; no cough. There was disinclination, amounting to inability, to take food; there was no albumen nor sugar in the urine; the blood was normal as to microscopical appearance; the pulse feeble, but regular. Steel wine and quinine were given once a day, and afterwards pepsine; the latter produced vomiting. She gradually, however, sank and died Dec. 4th. Nutrient enemata were attempted, but they produced so much distress that they could not be repeated.

*Dr. Wilkes' report of the post-mortem examination.*—Inspection twenty hours after death: the body was remarkably white. The brain was pale and watery; there was increased serum in the surface, and in the ventricles. Pleura: There was clear serum to the amount of several ounces in the chest. The lungs were very pale and watery. Heart: There were two or three ounces of serum in the pericardium; the heart was rather small, and there was an excess of fat seen on the front aspect; the valves were healthy; the excess of fat on the surface was seen to encroach on the muscular fibre, and to penetrate even to the interior, the walls being pale and soft. In the interior of the heart, especially in the left ventricle, well-marked fatty degeneration was seen; white zigzag striæ were present on the muscular columns and on other parts; the tissue was soft (the microscope confirmed this appearance of degeneration of the tissue); the coronary arteries were healthy. There was serum on the peritoneum; the stomach and liver were apparently quite healthy. The spleen was healthy, as were the kidneys and the supra-renal capsules.

The poor woman had resided in a damp and unhealthy locality, and, depending upon her needle for her livelihood, she had only obtained a scanty supply of the ordinary necessaries of life. The weakness of her state manifested itself first in the cessation of the menses at thirty-four years of age, and the catamenia did not again appear; her health, however, subsequently improved. The first marked symptom of disease consisted in gastric irritation, as shown by occasional attacks of vomiting, and these attacks took place at irregular intervals for several years; but for eight months the vomiting had become very severe, sometimes taking place immediately after the food had reached the stomach, and sometimes several hours elapsed; pain at the scrobiculus cordis and diarrhœa followed. She never suffered from hæmatemesis; and there was no evidence of organic disease of the chest, nor in the lymphatic glands, nor in the spleen, kidneys, uterus, liver, and supra-renal capsules; no trace of suppuration nor of disease of the spine could be found. Whilst in this prostrate condition, nine months before death, intense pain in the abdomen, with collapse, came on, resembling perforation of the intestine; but with rest and morphia she gradually rallied, and even improved so much as to be able to take animal food with an appetite. The symptoms passed off, and she left the hospital in improved health. After several months she was re-admitted with the same symptoms, but even more prostrate than at first. She was unable to take food, and the power of the heart and the force of the pulse gradually declined. Mineral acids, steel, quinine, stimulants, pepsine, nutrient diet, and enemata were all tried, and were either at once or after a short time set aside as intolerable by the patient. Life very slowly faded away, and death seemed to take place from mere atrophy.

The post-mortem inspection only revealed the effects of the atrophy, but did not indicate its cause. There was degeneration of the heart, and slight passive effusion into the pericardium as well as into the pleura and peritoneum; but no other organic change could be found. The blood had been examined microscopically during life, but without manifesting any excess of white corpuscles; and after death, no glandular disease could be traced.

Where, then, are we to look for the cause of this complaint, and what is its nature? The first symptoms indicate gastric irritation, and an interference with digestion; there was a deficiency in the quantity of nutriment absorbed,and hence atrophy. After death, however, no trace of organic change could be found in the stomach; it was reported as being in a healthy state—there was neither thickening nor cicatrix. It is to the nervous supply of the stomach that we must direct our attention; it was there that the fault originally commenced, and in that direction must we seek for the origin of the disease; and to combat that defective condition must be the aim of our treatment. In closely watching the gradual termination of these cases, we find that the lamp of life really dies out. The state has been called by some practitioners one of chronic syncope, for the patient is constantly closely bordering upon fainting; and the attempt to get out of bed in the extreme prostration may be followed by sudden death. There is diminished power in the heart, absence of the proper stimulus of healthy blood, and exhausted nervous power; in fact, it is a state of anæmia with one of asthenia.

In the treatment of these cases many patients, at an early stage, completely recover under the influence of bracing air, nutrient and stimulating diet, aided by the preparations of iron. The power of healthy assimilation is gradually restored, and the functions of the mind, as well as of the body, are performed in a vigorous manner; the red corpuscles of the blood increase in quantity, and with returning color we find restoration of health. Sometimes there is great advantage in combining quinine with the steel, and, if necessary, aloetic purgatives, or the extract of nux vomica, in small doses, may be added. Thus half a grain of the sulphate of iron, with one grain of quinine, one grain of the aloetic pills, and one grain of extract of henbane, taken twice a day after a meal, often agree exceedingly well; and better than when administered in a fluid state. In some instances, even small doses of the nux vomica have been followed by faintness, and I generally omit it altogether; and I have witnessed the same effect after very minute doses of strychnia.

Irritability of the stomach is a common symptom, and with difficulty checked; ice, bismuth, with soda, and chloric ether, minute doses of morphia and hydrocyanic acid, lime-water, the effervescent salines, carbonic acid, alone or in combination, may be used. Occasionally counter irritation at the serobiculus cordis gives some relief, but I am not partial to it as a remedy; nor have I found the same amount of benefit from the preparations of silver and creosote as in some other states of gastric irritation. Afterwards, dilute hydrochloric acid, with vegetable infusions, may be tried, not only as a tonic, but to facilitate the solution of food.

As diet, milk, with lime-water in small quantities, and often repeated, or mutton broth; arrow root, or other farinaceous food, may be also given, made with milk or with water; and, if there be great prostration, stimulants may be conjoined.—Nutrient enemata are certainly beneficial in many instances. Electricity, which in some cases of chlorosis has been followed by a speedy cure, is not, apparently, of any service in this more severe malady. Nor have I witnessed any benefit from pepsine. In the excessive anæmia and prostration which exist in such an instance as we have detailed, no remedy, however, is of any permanent benefit, and the patient very gradually, but with steady course, loses strength and power till life ceases from simple exhaustion. The stomach becomes, apparently, incapable of tolerating the presence of food, even when the patient can be persuaded to swallow it; so that the solution and absorption of it in sufficient quantity to sustain life are rendered almost impossible; and, in proportion as the nervous exhaustion increases, the morbid state of the stomach is also aggravated; the one reacts upon the other, each increasing the other's defect. In the exhaustion from over lactation, or from extreme mental distress, the same gastric inefficiency exists; there is the same complete loss of appetite, disinclination to take food, and, if swallowed, it will remain undissolved, and vomiting or faintness will follow; in the latter cases, however, the nervous exhaustion may be taken away, either by the removal of the cause of depression, or by the soothing effect of passing time and right treatment. Not so in the former; in them the nerve-strength and gastric power decline, *pari passu*, till they cease together.

Several other cases of anæmia are appended, different in kind, but each possessing great individual interest.

*Anæmia; diabetes insipidus; roseola; irritability of stomach; relief by Turkish bath.*—Henry T——, aged twenty-five, was admitted into Guy's Hospital, under my care, April 2nd, 1862. He was a spare man, married, and by trade a bricklayer; and some years ago he had had syphilis. Ten weeks before admission he had pain in his head, and his sight became confused, so that he was scarcely able to walk about; but the sight has been partially restored. Five weeks later he had thirst, and three weeks later still began to pass large quantities of urine. On admission, he was passing about a gallon of urine in the twenty-four hours; his skin was very dry and chippy; and his face had a red, patchy, and somewhat scaly appearance, as if he was convalescent from scarlet fever or erysipelas; there had, however, been no such illness. There was no sweetness of the breath, nor sponginess of the gums; the urine was pale and clear, and free from albumen and sugar. His appetite was very poor; the stomach irritable; the tongue clean; pulse very compressible. There was no evidence of any disease of the lungs or heart; the abdomen was supple and free from pain. He was ordered quinine.

On the 5th, the stomach was very irritable—food almost at once rejected; and finding that some years ago he had had syphilis, effervescing mixture with iodide of potassium was prescribed. Meat diet, with bread and vegetables was allowed.

On the 9th, the redness of the face was not general, but there were red and circular patches, slightly raised, and dusky in color; the redness disappeared on pressure, and the erythematous patches were almost continuous. The skin was roughened; occasionally, however, the skin became pale. The pulse was very compressible; the stomach still irritable; and the urine large in quantity (about ten pints), sp. gr. 1005. Soda-water and brandy were ordered.

16th.—There was less vomiting; the face was still in the same state; the skin very dry; the urine as before; but the patient said that he felt better.

On the 26th there was less prostration, but the general symptoms were the same.

On May 4th, violent sickness came on for several hours.

On the 10th he had a Turkish bath, which produced profuse perspiration and suppleness of the skin; the pulse became firmer, and his symptoms were all relieved. On the following day he continued better, and the face had lost its red and patchy appearance; and on the 12th he left the hospital greatly relieved. The urine, however, was still in considerable excess (six to eight pints), and of the same general character.

*Remarks.*—This case was an obscure one. Irritability of stomach, with polydipsia, and a dry desquamative condition of the skin, were the principal symptoms. The prostration was extreme; but saline effervescing medicine with iodide of potassium, and stimulants, afforded partial relief. The action of a Turkish bath in producing powerful diaphoresis certainly afforded most efficient help in the treatment of this patient, and greatly conduced to his recovery.

*Anæmia; bronzed skin; relieved; disease of the supra-renal capsules (?); periostitis.*—Eliza F——, aged forty-one, was admitted under my care into Guy's Hospital, April 9th, 1862.—She was a married woman, who had resided at the Lower-road near the Surrey-canal. Five years previously she had "bilious fever," when she had vomiting, but no purging, and was confined to her bed for six weeks. Three years before admission she vomited blood, but did not reject food. Till two years menstruation had been profuse; it then ceased altogether.—There had been no return of vomiting; but the prostration of strength had become extreme. For three weeks she had pain in the legs, shoulders, and back; pain came on across the chest; there was no vomiting, and the bowels were regular. On admission there was slight cough, and the pulse was very feeble and compressible. She was short in stature, the countenance was bronzed, and she so weak that she was scarcely able to stand. There was no evidence of disease of the heart or lungs; but slight tenderness at the serobiculus cordis on pressure.—She was ordered three ounces of brandy, and ten grains of the ammonio-citrate of iron was given in julep of ammonia three times a day, and nourishing diet allowed. The brandy was afterwards changed to wine. Her strength slowly returned; but on April 29th she complained of great pain in the left side. The periosteum was thickened and very tender, and the pain was more severe at night. There was melasma ab igne on the legs. The pulse was rather stronger. Three grains of iodide of potassium was given with the ammonia instead of the iron. The pain in the legs gradually ceased, the thickening disappeared, the color was less deep, and her strength returned, so that she was able to get about the wards and go into the hospital grounds. On May 27th she was desirous of going home, and left the hospital greatly relieved.

*Remarks.*—This patient had the bronzed complexion, with excessive prostration, and with previous irritability of the stomach; but the occurrence of periostitis, and the amount of relief afforded, not only to the pain and periosteal thickening but to the prostration and deep sallowness of the complexion, rendered it doubtful whether the disease did not principally consist in syphilitic cachexia.

*Anæmia following jaundice after gall-stone; purpurea hæmorrhagica; relieved.*—Mary S——, aged forty-five, was admitted into Guy's Hospital under my care April 22nd, 1862. She had been a widow for two years, and resided at Camden-town. Menstruation had ceased for six months. In December, 1861, after great mental anxiety, sudden pain came on across the abdomen, vomiting, and in a few days, jaundice.—These symptoms referred to gall-stone; and although the jaundice had almost gone when she came under my care, she had continued weak and prostrate. Seven weeks before admission pain in the back and at the stomach came on; she was then anæmic, slightly sallow and emaciated; she suffered from headache, and from pain at the region of the pylorus, brought on especially by solid food; food of that kind brought on severe pain, or was rejected; the bowels were constipated, and the evacuations pale; the tongue was slightly furred at the back; the heart was normal, but its action feeble; there was no disease of the chest, but the right side was more coarse than on the left; the abdomen was contracted; dullness in the right hypochondriac region extended nearly to the umbilicus, and the surface of the enlarged liver did not feel perfectly smooth; there were some enlarged glands in the neck on the right side, and in the right axilla; the urine was clear, and free from both bile and albumen. Dilute hydrochloric acid, with compound infusion of senna and of orange peel, being found to agree with the stomach, were substituted for the effervescing mixture; and, as an alternative aperient, one grain of podophyllin with two grains of extract of henbane, every morning. She seemed to improve in health, but on May 24th she stated that on the previous day dark spots suddenly appeared on the skin, with bleeding from the gums; a slight tingling sensation was produced in the skin; and while she watched the part spots of a greenish discoloration, equal in size to a half crown, gradually formed; these were on the arms and legs. Other small petechial spots were observed. There was semi-coagulated blood adhering to the gums and tongue, and about half a pint had been expectorated; the tongue was otherwise clean. The pulse was sharp and rather irritable; the bowels open; no blood in the motions nor in the urine; no headache; patient tolerably cheerful. She was ordered ice in the mouth, eight ounces of wine, and half a drachm of tincture of iron with two grains of quinine in water three times a day.

On May 26th the bleeding continued, but in a less degree; and there were numerous spots on the arms, &c.

27th.—Bleeding less, but the patient was prostrated. Greens and two ounces of brandy with ten ounces of wine, were given.

31st.—The bleeding returned, and she had a waxed countenance.

June 4th.—She was much blanched. To continue the same treatment.

7th.—Bleeding ceased for several days. Tongue clean; spots disappearing; pulse hæmorrhagic; countenance very pallid.

14th.—No return of bleeding; color returning. She feels stronger.

July 8th.—Steadily gaining strength. The symptoms greatly relieved, and no trace of the purpura.

*Remarks.*—The onset of this affection resembled that of gall-stone; but the continued enlargement of the liver, the pain at the pyloric region, and especially the enlargement of the glands in the neck and axilla, led me to fear organic disease. The sudden occurrence of purpura hæmorrhagica was remarkable; in half an hour bleeding into the skin and from the gums became very severe. She described the onset of the echymosis in the skin as being preceded by a smarting, pricking pain, and whilst she watched, discoloration took place.—The amount of blood lost was very great, and the patient became completely blanched. Stimulants were given very freely and tincture of iron with quinine administered every few hours. The symptoms slowly subsided, and the patient left the hospital anæmic, but her dyspeptic symptoms were greatly relieved. After a few months jaundice again came on, and she applied as an out-patient. The symptoms have again subsided, but the return of enlargement of the glands renewed the original suspicion of organic disease.—*London Lancet.*

